# Miscellaneous Items

**Published:** 1890-02

**Authors:** 


The deepest bore-hole in the world, claimed at different times for a number of places, is, according to latest accounts, at Schladebach, a small German village near Leipzig. It measures 1,748.4 meters or about 5,735 feet. The time expended in boring to this depth amounted to six years, at a cost of $52,500. A peculiar experience encountered in connection with this and other deep holes in different parts of Germany is, according to *Uhland's Wochenschrift*, that the observed temperatures, while steadily increasing with the depths, show a smaller ratio of increase in the lower strata.

At the last meeting of the Prussian Academy of Sciences, a large number of grants were made for scientific and medical purposes, amounting in all to 18,000 marks, or about $5,000. To Professor Brieger a grant of 1,800 marks ($500) was made to enable him to continue his investigations regarding the ptomaines, and to Dr. Fleischmann, of Erlangen, a like sum to enable him to procure material for his embryological researches. Many other grants were also made but were for biological or other scientific purposes.

Original Investigation.—The Vermont Microscopical Association has just announced that a prize of $250, given by the Wells & Richardson Company, the well-known chemists, will be paid to the first discoverer of a new disease germ. The wonderful discovery by Prefessor Kock, of the cholera germ, as the cause of cholera, stimulated great researches throughout the world, and it is believed this liberal prize, offered by a house of such standing, will greatly assist in the detection of micro-organisms that are the direct cause of disease and death. All who are interested in the subject, and the conditions of this prize, should write to C. Smith Boynton, secretary of the association, Burlington, Vt.

If you use a fountain pen, and find it difficult to unscrew the tip, wrap a rubber band a few times around it, and this will give you a good grip. A string or dampened piece of paper will also help.—*Med. and Surg. Reporter.*

Abscess of the Antrum.—A new diagnostic sign of abscess of the antrum was brought forward by Dr. T. Heryng, of Warsaw, at the Congress of Otology and Laryngology, held at Paris during September. The patient is placed in a dark room and his mouth lit up with a small electric lamp placed upon the tongue. Two bright red spots will then appear below the lower eyelids. If the cavities are filled up with pus, or occupied by a tumor, these red spots will not appear, but as soon as the pus escapes or the cavity is washed out the spots again become visible.

Alleged Danger in Wooden Toothpicks.—It has been assertes that chewing wooden toothpicks sometimes produces small ulcers in the mouth, and that even the stomach has been similarly affected by the action of the small particles of wood detached by chewing.—*Druggists' Circular.*

Fire Extinguisher.—Take 20 pounds of common salt and 10 pounds of sal ammoniac (chloride of ammonium) mix them and dissolve the mixture in 7 gallons of water, bottle and keep in each room to be used in case of fire by throwing the bottles with force into the fire so that they will be broken. The chlorine fumes of the liquid extinguishes the fire.—*Formulary and Drug. Magazine.*

A certain undertaker's energetic wife ekes out the family purse by keeping a boarding-house. The boarders claim to be able to tell with unerring accuracy when the husband has had charge of a funeral, because the next day there are flowers on table and ice on the butter.—*Tid Bits.*

Fruits, to do their best work as correctives for disordered digestion, should be eaten at the beginning of a meal, and not after oily foods—meats, vegetables, or high seasoning—when they become more of a curse than a blessing.

